# Can coffee prevent caries?

**DOI:** 10.4103/0972-0707.53336

**Published:** 2009

**Authors:** PC Anila Namboodiripad, Sumathi Kori

**Affiliations:** Department of Oral and Maxillofacial Pathology, KLE Society's Institute of Dental Sciences, Bangalore, Karnataka-22, India; 1 Department of Oral Medicine and Radiology, KLE Society's Institute of Dental Sciences, Bangalore, Karnataka-22, India

**Keywords:** Caries, coffee, DMFS score, milk, sugar, Streptococcus mutans

## Abstract

**Aim:**

To determine the anti-carious effect of coffee in humans. Coffee represents one of the most consumed products by the population.

**Materials and Methods:**

A random sample of 1000 individuals, of both sexes, who consumed only coffee as a beverage and who visited the Out-Patient Department of KLE Society's Institute of Dental Sciences, with a dental complaint and no history of any major illness, were considered as subjects. The patients' histories with regard to the coffee intake, such as, period of consumption, frequency of consumption, whether taken with milk or wihout milk, with sugar or without sugar, and the brand make, was noted. History of the type of diet, consumption of sweets, periodicity of brushing, and whether they had undergone fluoride applications were also noted. A thousand patients who consumed beverages other than coffee were taken as the control.

**Results:**

The results showed that coffee most consumed was roasted coffee, and the frequency on an average was about three cups per day, for an average period of 35 years. The Decayed/Missing/Filled Surface (DMFS) scores varied from 2.9, in subjects who drank black coffee, to 5.5 in subjects who consumed coffee together with sweeteners and creaming agents. The DMFS score was 3.4 in subjects who consumed coffee together with milk but no sugar. The DMFS score of the control subjects was 4, indicating that coffee if consumed alone had anticaries action, but in the presence of additives the antibacterial and anticaries action was totally minimized.

**Conclusion:**

Thus coffee can help in prevention of dental caries if consumed without additives.

## INTRODUCTION

*‘That the drinking of warm coffee and Chinese tea has further augmented the tendency of this period to a multitude of chronic diseases and thus aided psora. I least of all can doubt, as I have made prominent, perhaps too prominent, the part which coffee takes with respect to the bodily and mental sufferings of humanity, in my little work on the ‘Effects of Coffee’ (Leipzig, 1803). This perhaps undue prominence given was owing to the fact that I had not then as yet discovered the chief source of chronic disease in Psora.’ (Chronic Diseases, Jain Edition, 1978, (Tafel translation of 1896), Vol 1, pp13-14). *However, on further evaluation by the author it was found to mean, “In such a situation coffee can be safely used to dampen and neutralize the activity of the psoric miasm in a positive way.”[1] 

Coffee brews are very widely consumed in the world due to their pleasant flavor and taste as well as for their stimulating effect on the mental and physical activities. Mixed messages from scientists about the advantages or disadvantages of coffee are almost a weekly event.[2]Research into the benefits and disadvantages of coffee, shows that scientists claim that the drink either protects the arteries or damages them, that it contains many helpful antioxidants, or that it can cause anything from cancer to bone loss. On the other hand it decreases the risk of many diseases like diabetes, Parkinson's diseases, colon cancer, and many more according to some recently published studies.

It elevates mood and can treat severe headaches. If coffee is drunk in limited amounts it can help in having healthier and whiter teeth as well as enjoying other health benefits. Besides keeping one alert and awake, coffee has been linked to an increasing number of potential health benefits. Coffee provides more than just a morning jolt and has been found to contain substances called antioxidants, which are beneficial to the human body. Several years ago, in California, a research conducted by Takayuki Shibamoto, a professor of environmental toxicology, found that freshly brewed coffee contains potent antioxidants equal to the amount found in three oranges. Antioxidants in general have been linked to a number of potential health benefits, including protection against heart disease and cancer, protection against liver and colon cancer, type 2 diabetes, and Parkinson's disease. The study analyzed the antioxidant contents of more than 100 different food items, including vegetables, fruits, nuts, spices, oils, and common beverages and found out that coffee had a good amount of primary antioxidants. Of all the foods and beverages studied, dates actually had the most antioxidants, based solely on serving size. However, since dates are not consumed at anywhere near the level of coffee, the latter tops the food analysis on the combined basis of both antioxidants per serving size and frequency of consumption. The antioxidant in coffee is called methylpyridinium. Oddly enough, this tongue-twister chemical is not found in large amounts in other foods. Even odder, it is not present in the raw coffee bean. It is created during the roasting process from the trigonelline that is present in raw coffee beans.[3]Two similar studies, done by Dutch and also Harvard scientists in 2004, indicate that by raising the overall metabolism, other aspects, like fat oxidation and the mobilization of glycogen in muscles may be stimulated as well. The caffeine in coffee is known to affect the manner in which the body handles sugar absorption: A few cups of coffee a day can greatly reduce the onset of type II or adult-onset diabetes. Coffee also contains substantial amounts of potassium, niacin, magnesium, and important antioxidants such as tocopherols and phenol chlorogenic acid. It is also been known to thwart migraine headaches, (the results show that both green and roasted coffee possess antiradical activity, and their more active component is 5-O-caffeoyl-quinic acid. Moreover the roasting process induces Maillard reaction products, such as, melanoidins, which also possess antiradical activity, in coffee. These results could explain the neuroprotective effects found for coffee consumption in recent epidemiological studies)[4]and to ease pain and relieve symptoms associated with asthma. Some beverages and foods protect tooth surfaces against Streptococcus mutans colonization. Fruit juices and beverages inhibit the glucosyltransferases in the * Streptococcus mutans*. Adhesion of * S. mutans* to the tooth surface, which occurs due to this glucosyltransferase, is a crucial step in the initiation and development of dental caries. A new research study indicates that coffee might help prevent cavities.[5]The finding is reported in the Journal of Agricultural and Food Chemistry,[6]a publication of the American Chemical Society. A new research published shows that coffee made from roasted coffee beans has antibacterial activities against certain microorganisms. Both Gram-positive and Gram-negative bacteria, including * S. mutans are* a major cause of dental caries.[2]Inhibition by cocoa, coffee, and tea is due partly to gelatin-precipitable tannins and partly to components that exhibit properties of monomeric polyphenols. Catechin, a known constituent of these beverages, is an effective inhibitor of the enzymes. The effects of fruit juices are attributable mainly to the inhibition of the glucosyltransferases, by the endogenous fructose and glucose. The findings show that the naturally occurring constituents of foods can inhibit extracellular polysaccharide formation from sucrose. Such constituents may play a role in regulating dental plaque formation * in vivo* and, thereby, have long-term effects on the development of dental caries. 

Coffee beverages are very complex mixtures of several hundred chemicals that either occur naturally, or else, are later induced in coffee by the roasting process, that is, in the form of nicotinic acid or melanoidins. The occurrence of such a great number of chemicals in coffee has recently induced many researchers to investigate other coffee bioactivities. Coffee, in particular roasted coffee, has been found to act as a potent antioxidant and to inhibit lipid peroxidation both in the chemical and biological systems in rat liver microsomal fractions.[6]

Scientists at two Italian universities conducted laboratory tests which showed that coffee molecules prevented adhesion of * S. mutans *on tooth enamel. The lead author, Gabriella Gazzani, who is a professor in the Department of Druggist Chemistry at the Pavia University, stated that all coffee solutions have high antiadhesive properties due to naturally occurring and roasting-induced molecules. She and researchers at the University of Ancona analyzed the sampled green and roasted Arabica and Robusta coffee from different countries, and all the tested samples inhibited * S. mutans* adsorption and showed inhibitory activity ranging from 40.5 to 98.1%.[2]It was also found that all green (unroasted beans) coffee samples were less active than the corresponding roasted coffees.[5]The roasted coffee also has antiadhesive properties. In this way it prevents adhesion of * S. mutans* and other harmful material to the teeth. Regular coffee drinkers not only have caries-free teeth, but also their teeth are whiter as compared to others. 

 The researchers examined caffeine and non-caffeine samples of ground and instant coffee. Instant coffee had a higher level of inhibitory activity against * S. mutans*.[7]As for caffeine and decaf, the results seemed to indicate that caffeine was not involved in the antiadhesive properties of coffee solutions.

 It is suggested that trigonelline, a water-soluble compound in coffee that contributes to the aroma and flavor of the beverage, may be responsible for the coffee′s antiadhesive activity. In the absence of animal model data, caution is advised in the interpretation of the * in vivo* significance of these results.[2]Melanoidin was another reason for this antiadhesive action,[3]reported Science News.

Besides other popular antioxidant sources such as tea, milk, chocolate, bananas, and dry beans, this wonder bean has outranked corn and cranberries. Although the findings would seem to encourage people to go out and drink more coffee, moderation is necessary. In addition, fruits and vegetables are on the hit list because they are better, from an overall nutritional point of view, owing to their higher content of vitamins, minerals, and fiber.[8] 

## MATERIALS AND METHODS

One thousand subjects who visited the Outpatient Department of Oral Medicine and Radiology, of the KLE Society's Institute of Dental Sciences, Bangalore, who consumed coffee, were picked randomly and they comprised of both sexes, males and females, and 1000 subjects who consumed other liquid drinks (controls) were taken as the control. Individuals who suffered from systemic ailments such as diabetes, heart problems, and any immunocompromised states were excluded from the study. Factors which altered the DMFS scores were also taken, such as, consumption of sweets, diet — whether vegetarian or non-vegetarian, number of times the teeth were brushed, brushing techniques, frequency of visits to a dentist etc. The subjects' average age was 35 years. History of coffee habit such as frequency of consumption/day, and the duration of the consumption in years were noted. Details of the method of consumption were enquired about, such as, whether the coffee was consumed alone or in combination with milk and sugar. The findings were recorded and tabulated. The DMFTS score was calculated. A single examiner examined all the subjects.

In this study, * P *value** ≤ 0.05.

The C indicates coffee, M indicates milk, S indicates sugar, N is the number of subjects who consume coffee, age is the average age of subjects, frequency is the consumption of coffee in a single day, duration is the average period for which coffee has been consumed and DMFS represents Decayed, Missing, Filled, Teeth, and S represents the surfaces involved.

## RESULTS

**Figure 1:**
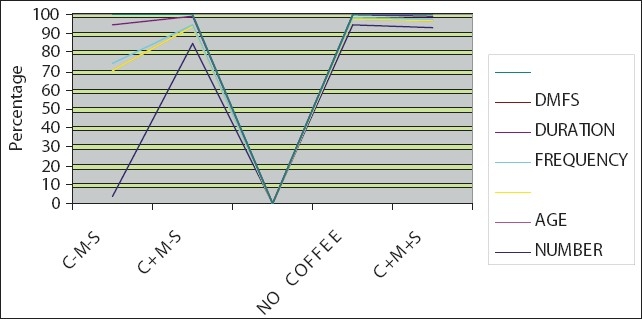
Coffee and its role in dental caries

A one way ANOVA TEST showed a total mean of 13.65, standard deviation of 4.682, and a degree of freedom of 3. A * p* value ≤ 0.05 was considered. The results were statistically significant [Table 1 and Figure 1].

**Table 1:** The mean and standard deviations have been tabulated for a * p* value of ≤ 0.05

	C − M − S	C + M − S	C + M + S	Other beverages
Number/1000	2	324	674	1000
Age	38.2 ± 2.7100	36.6 ± 3.8427	31.6 ± 4.892	34.81 ± 6778
Frequency	2.2 ± 1.229	2.4 ± 1.074	1.8 ± 788	2.6 ± 1.173
Duration	11.7 ± 3.772	16.3 ± 3.713	13 ± 2.625	14.6 ± 3.864
DMFS	2.9 ± 1.972	3.4 ± 1.505	5.5 ± 1.957	4 ± 2.1602

## DISCUSION

Dental caries is one of the scourges of man, which appears to be exacerbated rather than ameliorated by general improvements in living standards. The onset of caries is a phenomenon of the tooth surface and is initiated by the secretion of metabolic products of microorganisms in the dental plaque, which adhere to the enamel surface, particularly in those areas which are least self-cleansing. There is much evidence to show that the dental plaque can produce organic acids from the metabolism of fermentable carbohydrates, and it is these acids, which by lowering the pH locally, cause the initial breach in the highly mineralized enamel. Dental caries is thus primarily of an environmental origin; however, its onset and rate of progression may be influenced to a considerable degree by the chemical and physical structure of the enamel surface.[8]

The * Coffee Statistics Report* also states that coffee is the most popular beverage worldwide, with more than 400 billion cups consumed each year. Over the years, some 19,000 studies have looked at the health impact of coffee. "Overall, the research shows that coffee is far more healthful than it is harmful," Tomas DePaulis, PhD, a research scientist at Vanderbilt University′s Institute for Coffee Studies, tells WebMd.[7]The Institute for Coffee Studies (ICS) was recently established at Vanderbilt University Medical Center, Nashville, USA, for the study of the health benefits of coffee. ICS is dedicated to investigating the chemical nature of coffee and exploring new therapeutic uses of coffee by the systematic evaluation of specific constituents found in roasted coffee using the latest technologies in cell physiology, receptor pharmacology, molecular biology, medicinal chemistry, and genetics.[9] 

Few of the significant bioactivities of coffee documented are antioxidant activity, anticarcinogenic activity, antimutagenic activity, and so on. Various compounds responsible for the chemoprotective effects of coffee are mainly polyphenols, including chlorogenic acids and their degradation products. Others include caffeine, kahweol, cafestol, and other phenolics. Coffee also shows protective or adverse effects on various systems like the skeletal (bone) system, the reproductive system, the nervous system, the cardiovascular system, the homocysteine levels, the cholesterol levels, and so on.[10]According to recent studies, coffee is beneficial for our teeth. Coffee that is made from roasted beans has antibacterial activity. Coffee is active against * Streptococcus Mutans*, the organism causing dental caries. Roasted coffee also has antiadhesive properties. In this manner, it prevents adhesion of * S.mutans* and other harmful material to the teeth.[4]Regular coffee drinkers not only have caries-free teeth, but also their teeth are whiter as compared to others. Non-roasted coffee does not have such a strong antibacterial activity.[12]A study shows that each cup of coffee has a large amount of antioxidant polyphenols in its roasted form [Maximum antioxidant activity was observed for the medium-roasted coffee; the dark coffee had a lower antioxidant activity despite the increase in color.[11]That is not diminished by adding milk or by decaffeination, which is totally contradictory to our study where the addition of sugar and milk greatly enhanced the DMFS scores. Thirty-five percent of coffee drinkers drink their coffee black, while 62% add a sweetener and/or a creaming agent. This finding could be corroborated by our study, where only 2% had black coffee as compared to 98.0% who added sweeteners to their coffee. Coffee contains similar levels of antioxidants as black tea, which can be just as beneficial to health, according to Scottish scientists, who have found that coffee′s antioxidants are highly absorbable by the human body. 

The antioxidant activity as determined by the lag time was in the range of 292 – 948 minutes for coffee, 217 – 444 minutes for cocoa, 186 – 338 minutes for green tea, 67 – 277 minutes for black tea, and 6 – 78 minutes for herbal tea. Addition of milk did not alter the antioxidant activity.[9]It is also clear that roasting coffee dramatically increases its total antioxidant activity, so other compounds, which are formed during roasting, such as melanoidins, may also have an important role to play. Melanoidins contribute to the strong antioxidant properties of coffee and in the development of coffee aroma during the coffee roasting process. However, roasting also reduces the trigonelline to niacin, and hence, the antiadhesive action of coffee on * Streptococcus mutans* is reduced.

The clinical data from our study showed that the frequency of consumption was uniform throughout the world and was found to be three times in a day. In our control, that is, subjects who did not consume coffee, the DMFS score was 4. DMFS scores of those who consumed coffee, varied from 2.9, where coffee was consumed without sugar and milk and 5.5 when it was taken with milk and sugar indicating that coffee has anticaries action, but the addition of the sweeteners and creaming agents, reduces the antibacterial and anticaries action of coffee. 

## CONCLUSION

Tea, particularly green tea, has received widespread media coverage for studies revealing its health benefits, but Professor Alan Crosier from the University of Glasgow says that the new findings show that coffee can also be considered a valuable source of antioxidants, supplementing those obtained from fruit and vegetables. Research has already established the antioxidant activity of coffee and confirmed its antioxidant component – chlorogenic acid, also found in fruits, such as apples. However, Professor Crosier's research demonstrated for the first time the high bioavailability of such antioxidants when consumed in coffee.[11]

“Black coffee must be strong and very hot; if strong coffee does not agree with you, do not drink black coffee. And if you do not drink black coffee, do not drink any coffee at all.” ~ * Andre Simon*
